# Osteogenic effect of magnesium oxychloride cement modified with phytic acid and loaded with strontium ranelate

**DOI:** 10.1186/s40824-023-00474-8

**Published:** 2023-12-10

**Authors:** Tingting Ma, Yijia Guan, Jinlun Feng, Yue Yang, Junying Chen, Wenjie Guo, Jianguo Liao, Yanru Zhang

**Affiliations:** 1 Henan Key Laboratory of Materials on Deep-Earth Engineering, School of Materials Science and Engineering, Henan Polytechnic University, Jiaozuo, China; 2 School of Medicine, Henan Polytechnic University, Jiaozuo, China

**Keywords:** Magnesium oxychloride cement, Phytic acid, Strontium ranelate, Water resistance, Osteogenic property

## Abstract

**Background:**

Magnesium oxychloride cement has good mechanical properties, but poor water resistance.

**Methods:**

Phytic acid, which can form chelate with Mg^2+^, was used to modify magnesium oxychloride cement, and the effects of phytic acid on the strength, in vitro degradation and biological activity of magnesium oxychloride cement were studied. Based on the preparation of phytic acid modified magnesium oxychloride cement with good water resistance and biological activity, osteoporosis treatment strontium ranelate was loaded on phytic acid- magnesium oxychloride cement, strontium ranelate/phytic acid-magnesium oxychloride cement was prepared.

**Results:**

It was found that the compressive strength of 1.25 wt% phytic acid-magnesium oxychloride cement after soaking in SBF for 28 d could reach 40.5 ± 2.0 MPa, 13.33% higher than that of the control group (when phytic acid was 0 wt%), and the mass loss rate of all ages was lower than that of the control group. The water resistance of magnesium oxychloride cement was effectively improved by phytic acid. After loading with strontium ranelate, the water resistance of 1.25 wt% phytic acid-magnesium oxychloride cement was improved. Cell experiments showed that strontium ranelate could effectively promote cell proliferation and improve the expression of osteoblast-related proteins. When strontium ranelate/phytic acid-magnesium oxychloride cement samples were implanted subcutaneously in rats for 4 w, no obvious inflammatory response was observed, and the material was tightly bound to the surrounding tissues. When bone cement was implanted into rat femur for 4 w, the bone cement was gradually wrapped and absorbed by new bone tissue, which grew from the outside to the inside, indicating that the bone cement containing strontium ranelate/phytic acid-magnesium oxychloride cement had excellent bone-forming ability.

**Conclusions:**

In conclusion, the results indicated that strontium ranelate/phytic acid-magnesium oxychloride cement composite bone cement had a potential application prospect in clinical bone repair.

**Graphical Abstract:**

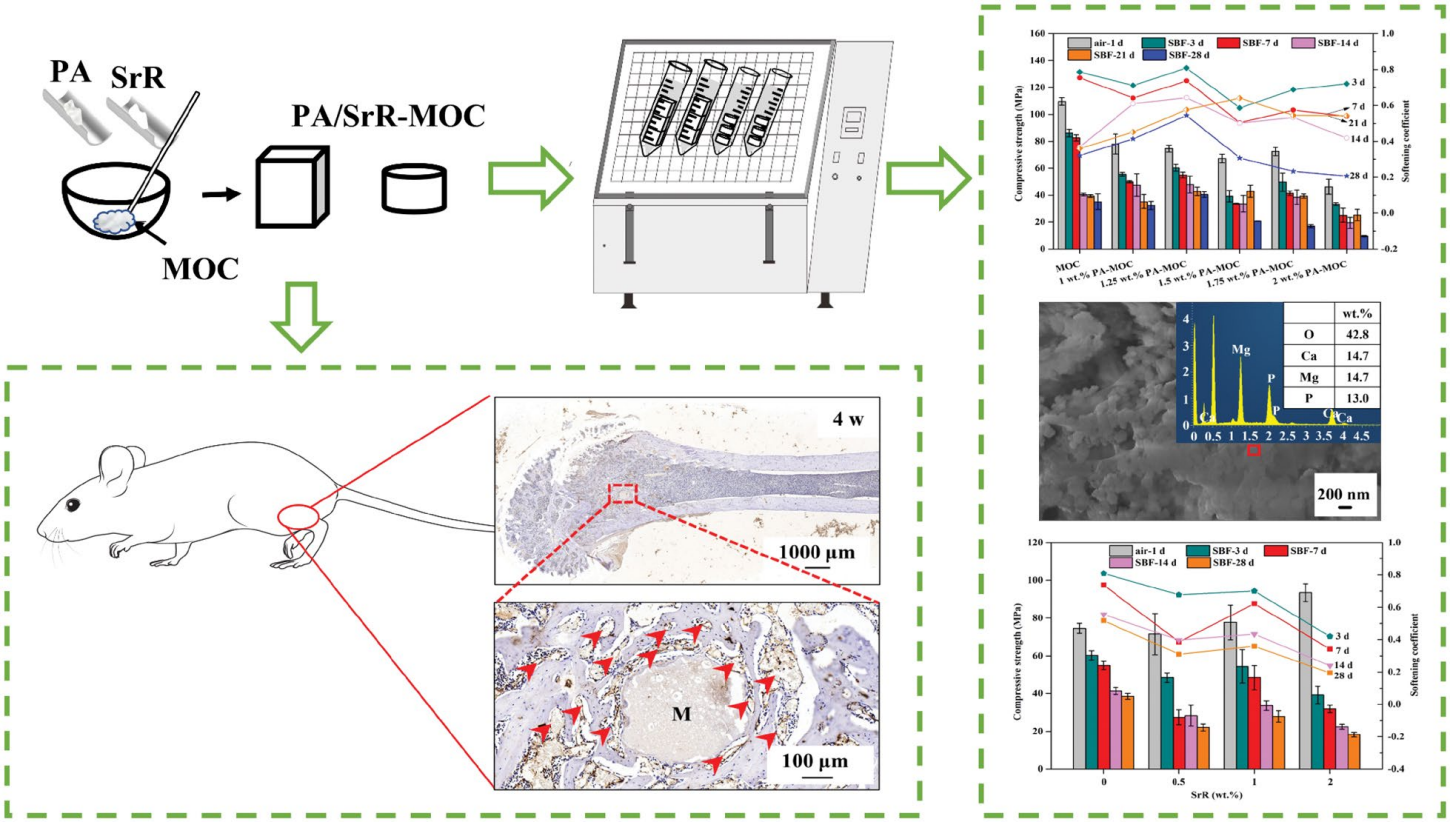

## **Introduction**

Magnesium oxychloride cement (MOC) is made from a system of MgO-MgCl_2_-H_2_O composed of magnesium oxide (MgO) and magnesium chloride (MgCl_2_) solutions through chemical reactions. At room temperature, the reaction of MgO and MgCl_2_ solution will form 3Mg(OH)_2_·MgCl_2_·8H_2_O (phase 3) and 5Mg(OH)_2_·MgCl_2_·8H_2_O (phase 5) [1]. Phase 5 is metastable and phase 3 is stable. Metastable phase 5 tends to transform into stable phase 3, but the strength and water resistance of phase 5 are higher than that of phase 3. Therefore, phase 5 is the phase that contributes the most to the strength of MOC [2].

MOC has advantages such as high mechanical strength, good cohesiveness and low cytotoxicity, so some scholars have proposed its application in the field of bone repair [3]. However, MOC has poor water resistance, and its hydration product phase 5 will gradually hydrolyze to form Mg(OH)_2_ in water [3], which reduces the compressive strength. Studies have found that to improve the water resistance of MOC, in addition to improving the water resistance by optimizing the composition ratio of MgO, MgCl_2_ and H_2_O [4], increasing the number of phase 5 crystals and reducing the formation of phase 3 and other hydration products, the structural state of MOC can also be improved by adding mineral admixtures, organic or inorganic admixtures to improve its water resistance. It was found that soluble sulfate [5], phosphoric acid and phosphate [6] and organic weak acid [7] could effectively improve the water resistance of MOC. Deng et al. [6] studied the addition of water-soluble phosphate to MOC slurry. The addition of phosphate reduced the minimum Mg^2+^ concentration required for the formation of phase 5, increased the content of phase 5, and significantly improved the water resistance of MOC. Li et al. [5] added ferrous sulfate (FeSO_4_) into MOC to change the pore structure of MOC. After soaking in 50ºC water for 48 and 96 h, compared with MOC, the compressive strength retention rate increased by 150.5% and 135.5%, respectively.

Phytic acid (PA, C_6_H_18_O_24_P_6_), also known as inositol hexaphosphate [8], is a phosphate-containing organic acid compound, colorless or yellowish paste liquid, extracted from plant seeds, grains or fruit shells. PA is easy to bind with polyvalent metal cations (such as Ca^2+^, Mg^2+^) through chelation [9–12]. Hurle et al. [9] modified calcium phosphate cement with PA and found that the chelation of phosphate groups contained in PA with Ca^2+^ could delay the setting time and improve its injectivity. The study of Meininger et al. [10] showed that the chelation of PA and Ca^2+^ improved the mechanical properties of dicalcium phosphate cement, and had a higher ability to promote cell proliferation. Liu et al. [12] prepared PA/Ca^2+^ conversion membrane on the surface of Mg-Sr alloy by using the strong chelating force of PA and self-assembly layer by layer. The study showed that the PA/Ca^2+^ conversion coating promoted cell proliferation because its surface composition contained phosphoric acid groups and Ca^2+^. Ye et al. [11] modified MOC with PA. After soaking the prepared samples in water for 7 d, the compressive strength of all PA-MOC samples decreased slightly, and the softening coefficient of PA-MOC composite material increased by more than 95.5% compared with MOC. This is because after PA was added to MgO-MgCl_2_-H_2_O ternary system, Mg^2+^ and phosphate anions in PA formed a hybrid network through chelation. In this network, the multiple phosphate anions of PA provided binding sites for Mg^2+^, and these sites could be cross-linked with multi-nuclear complexes to produce organic-inorganic copolymers. Due to its rigid tension, coordination bond and waterproofing mechanism similar to sandcastle worm, the copolymer had better water resistance.

Strontium ranilate (SrR) is a kind of osteoporosis treatment drug, which can not only inhibit bone resorption but also effectively promote bone regeneration [13]. On the one hand, SrR enhances the activity of alkaline phosphatase (ALP) and promotes osteogenesis by promoting the expression of osteoblast markers such as bone salivariin and osteocalcin [14]. On the other hand, SrR inhibits osteoclast activity and survival through activation of OPG/RANKL/RANK, NF-κB and other signaling pathways [15–18]. However, systemic administration of SrR can increase the risk of cardiovascular disease [19]. Compared with systemic administration, the control of local release of SrR can improve drug efficacy and duration of action and reduce drug toxicity.

Based on this, this paper added PA into MOC to improve the strength and bioactivity of MOC system, and on the basis of the optimal amount of PA, loaded SrR onto it, and studied the changes of mechanical strength, degradation performance and bioactivity of PA-MOC after the loading of SrR.

## Materials and methods

### Materials

Light burning magnesium oxide (MgO): The magnesium hydroxide (Mg(OH)_2_, analytically pure, Tianjin Guangfu Fine Chemical Research Institute, China) was calcined at 500ºC for 3 h, then cooled and sealed for use. Magnesium chloride (MgCl_2_·6H_2_O), analytically pure, Tianjin Kemiou Chemical Reagent Co., LTD., China. Potassium dihydrogen phosphate (KH_2_PO_4_), analytically pure, Tianjin Hedong District Hongyan Reagent Company, China. Phytic acid (C_6_H_18_O_24_P_6_, PA), 50% aqueous solution, Shanghai Aladdin Biochemical Technology Co., LTD., China. Simulated body fluid (SBF) was prepared according to the literature [20]. Deionized water was made in the laboratory.

### Preparation of PA-MOC and SrR/PA-MOC samples

#### Preparation of PA-MOC samples

The basic molar mixture ratio of MOC was n (MgO) ∶n (MgCl_2_) ∶n (H_2_O) = 8∶1∶13, the content of KH_2_PO_4_ in MOC system was determined to be 2 wt% according to the literature [21]. Each raw material was weighed according to the ratio in Table 1, prepared aqueous solution with different PA content, then dissolved MgCl_2_ in aqueous solution of PA, after fully dissolved, added KH_2_PO_4_ and stirred until no particles, finally added MgO powder and stirred evenly. The mixed slurry was injected into moulds of 10 mm ×10 mm ×10 mm and Φ10 mm × 2 mm. After curing for 24 h in a constant temperature and humidity incubator (temperature 37ºC, humidity 50%), PA-MOC samples were obtained.

**Table 1:** Detailed mixture proportions of PA-MOC

Group	PA (wt%)	KH_2_PO_4_ (wt%)	n (MgO)∶n (MgCl_2_)∶n (H_2_O)	liquid phase(aq)
MOC	0	2	8∶1∶13	H_2_O
1 wt% PA-MOC	1	2	8∶1∶13	H_2_O
1.25 wt% PA-MOC	1.25	2	8∶1∶13	H_2_O
1.5 wt% PA-MOC	1.5	2	8∶1∶13	H_2_O
1.75 wt% PA-MOC	1.75	2	8∶1∶13	H_2_O
2 wt% PA-MOC	2	2	8∶1∶13	H_2_O

#### Preparation of SrR/PA-MOC samples

By testing the strength, softening coefficient and mass loss of PA-MOC, the group with better performance was obtained as the benchmark ratio. The incorporation amounts of SrR were 0.5 wt%, 1 wt% and 2 wt% of the solid mass (MgO, MgCl_2_ and KH_2_PO_4_), respectively. During preparation, SrR was firstly dissolved in PA aqueous solution, and the other steps were the same as the PA-MOC preparation in Section "Preparation of PA-MOC samples".

### Degradation behavior of PA-MOC and SrR/PA-MOC in vitro

#### Compressive strength and softening factor

Part of PA-MOC and SrR/PA-MOC (10 mm × 10 mm × 10 mm) samples (6 samples in each group) after demoulding were tested by universal testing machine (CMT-20, Jinan Union Testing Technology Co., LTD., the head traveling speed was 1 mm·min^− 1^) and the average compressive strength was recorded as R_a_; The other part of the samples (6 samples per group and per cycle) were soaked in 50 mL polyethylene tube containing SBF solution. The volume ratio of the sample to SBF was 1∶10. Then the polyethylene tube was placed in a constant temperature water bath oscillating chamber (37ºC, 50 r/min oscillation frequency). After soaking for 3 d, 7 d, 14 d and 28 d, the samples were taken out, the surface moisture of the samples was dried, and the compressive strength of the samples was tested by universal testing machine. The mean value was R_(s,n)_, and the softening coefficient I_f_ of the samples was calculated according to formula (1) to evaluate the water resistance of the samples.

1 $${I}_{f}\text{=}\frac{{R}_{(s,\,n)}}{{R}_{a}}$$

#### Mass loss rate

The PA-MOC and SrR/PA-MOC round sheet samples (Φ10 mm × 2 mm, 6 samples in each group) were rinsed with absolute ethanol and dried under vacuum at 60ºC for 3 h, recorded its mass as M_0_, then put the sample into a 5 mL polyethylene tube and added SBF solution according to the ratio of sample to soaking liquid 1∶10. Then the polyethylene tube was placed in a constant temperature water bath oscillating chamber (37ºC, oscillation frequency 50 r/min), and the SBF solution was replaced every 3 d. The samples were taken out after soaking for 3 d, 7 d, 14 d, 21 d and 28 d respectively. After washing with absolute ethanol, the samples were dried under vacuum at 60ºC for 3 h. Weighed its mass and recorded it as M_t_; The mass loss rate M_L_ of the sample was:

2 $${M}_{L}\text{=}\frac{{M}_{0}-{M}_{t}}{{M}_{0}}\times100\%$$

#### *In vitro* ion concentration

The PA-MOC samples were placed in a polyethylene tube, and SBF solution was added according to the volume ratio of PA-MOC to SBF at 1∶20. The solution was soaked for 7 d, and the SBF solution was not changed during the soaking. 2 mL of the sample soaking solution was taken every day, and the samples were processed by automatic titrator (T960, Ji ‘an Energy Instrument Co., LTD., China) to determine the concentrations of Mg^2+^ and Ca^2+^ in solution.

#### The change of pH value

PA-MOC samples were soaked in 50 mL polyethylene tube containing SBF, and SBF solution was added in accordance with the volume ratio of PA-MOC to SBF of 1∶10. The samples were placed in a constant temperature water bath oscillating chamber at 37ºC with an oscillating frequency of 50 r/min. The SBF solution was replaced every 24 h. The pH meter (PHS-3E, Leici-Shanghai Yidian Scientific Instrument Co., LTD., China) was used to test the pH value of samples after soaking for different ages.

### Microstructure analysis of PA-MOC and SrR/PA-MOC

Fourier transform infrared spectrometer (FT-IR, Bucks HP9 2FX, PerkinElmer, USA), X-ray diffractometer (XRD, SmartLab, 9 kW, Rigaku, Japan) and field emission scanning electron microscope (SEM, Merlin Compact, Carl Zeiss NTS GmbH, Germany) were used to characterize the functional groups, crystal phase structure and microstructure of the samples.

### Cell experiment

#### Preparation of the extract

After sterilizing the Φ10 mm×2 mm round sheet samples with ultraviolet lamp for 12 h, according to the international standard ISO 10,993 Part 12, the sample was added to the complete culture medium immersion ratio of 1.25 cm^2^/mL. After soaking at room temperature for 24 h, the sample was filtered by 0.22 μm needle filter. Extract of materials (experimental group) was obtained, and the conventional complete medium without sample extract was set as the control group.

#### Cell culture

Mouse bone marrow mesenchymal stem cells (BMSCs), Shanghai Zhongqiao Xinzhou Biotechnology Co., LTD., China. To get complete medium for cell blast culture, the low-sugar medium (D5546, Sigma Aldrich Trading Co., LTD., Shanghai, China) was supplemented with 10% fetal bovine serum (04-001-1 A, Kailaibo Biotechnology Co., LTD., Changchun, China), 1% L-glutamine (G7513, Sigma Aldrich Trading Co., LTD., Shanghai, China) and 1% penicillin-streptomycin (double antibody) (R20016, Yuanye Biotechnology Co., LTD., Shanghai, China). BMSCs were cultured at 37ºC in a 5% CO_2_ incubator (HH.CP-TIN, Shanghai Qixin Scientific Instrument Co., LTD.). The complete medium was replaced every 2 d, and the cell growth was observed by inverted fluorescence microscope (DHXY-2, Shanghai Dylan Optical Instrument Co., LTD.).

#### Cell viability

Cell proliferation activity was detected on the third day and on the seventh day and CCK-8 (Cell-counting Kit-8) test kits (C6005M, Shanghai Biyuntian Biotechnology Co., LTD.) was used to test. 100 µL cell suspension was added into the 96-well plate at the concentration of 3 × 10^4^ cells /mL, and the medium was removed after 24 h. 100 µL extract was added into the corresponding well for the experimental group and complete medium for the control group. Five multiple wells were set in each group, and the corresponding extract and complete medium for each group were replaced every 48 h. After 3 and 7 d of culture, 10 µL CCK-8 working solution was added to each well and incubated in a CO_2_ incubator for 4 h under the protection of light. Then the 96-well plates were taken out. The absorbance values (OD values) corresponding to the 450 nm wavelength of the experimental group and the control group were detected using an enzyme label (Beijing Pulang New Technology Co., LTD.), each group of samples set 5 multiple holes and the data were processed.

#### Detection of ALP activity

1.5 mL 3 × 10^4^ cells/mL cell suspension was added into the 6-well plate for cell planking. After culturing for 24 h, the medium was removed, and 1.5 mL sample extract or control medium was added into the corresponding hole for culture in an incubator. Fresh medium for each group was replaced every 2 d. After culturing for 7 and 14 d, the medium was removed and washed twice with cold PBS solution. The 6-well plate was placed on the ice, and 200 µL cell lysate (P0013B, Biyuntian Biotechnology Co., LTD.) was added to each well drop by drop with a pipette gun. The lysate was blown several times with the gun to make full contact with the cells. After complete cracking, the cracked cells were collected into a centrifuge tube with a cell scraper and centrifuged at 13,000 rpm for 5 min using a desktop high-speed frozen microcentrifuge (DLAB Company). The supernatant was taken to obtain the total protein.

ALP concentration was determined by alkaline phosphatase assay kit (P0321S, Biyuntian Biotechnology Co., LTD.). Total protein concentration was determined with BCA kit (P0010, Biyuntian Biotechnology Co., LTD.). The OD value of ALP at 405 nm and OD value of total protein at 578 nm were measured by enzyme label, each group of samples set 5 multiple holes and the ratio of OD value of ALP to OD value of total protein was used as the measurement standard of ALP activity.

#### Staining of living and dead cells

Calcein/PI cell activity and cytotoxicity assay kit (C2015M, Biyuntian Biotechnology Co., LTD.) was used for staining of live and dead cells. The cells were inoculated in 96-well plates for 24 h, then the culture medium was discarded and washed with PBS for 1 or 2 times. 100 µL of Calcein AM/PI working solution was added to each well, and incubated for 30 min at 37ºC without light. After incubation, the staining effect was observed under fluorescence microscope (Calcein AM was green fluorescence, Ex/Em = 494/517 nm; PI is red fluorescence, Ex/Em = 535/617nm).

## Animal experiment

### Subcutaneous implantation experiment

Samples with the size of Φ5 mm×2 mm were prepared and sterilized by ultraviolet irradiation for use. Female SD rats weighing 200 ~ 250 g (3 samples per group) were selected for intraperitoneal injection with 10.0 wt% chloral hydrate solution. After anesthesia, skin incision of 1 cm parallel to the spine was made in a symmetric position on both sides of the spine. 3 d after surgery, the animals were fed normally after intramuscular injection of penicillin every day. The animals were killed at 4 w after surgery, and the subcutaneous materials and surrounding tissues were fixed in 4% paraformaldehyde for H&E, Masson and F4/80 immunohistochemical staining, respectively.

#### Femoral defect implantation experiment

Female SD rats with body weight of 200 ~ 250 g (3 samples per group) were selected. The rats were fasted for 1 d before surgery, and the surgical instruments were autoclaved at high temperature. The rats were injected intraperitoneally with 10.0 wt% chloral hydrate solution. The rats were observed after the injection. After the rats were fully anesthetized, the operation area was shaved and placed on the operating board in prone position. A trephine was used to establish a Φ0.8 mm × 3 mm femur defect inside the femoral condyle, and the muscle and skin wounds were sutured successively. 3 d after surgery, the animals were given intramuscular injection of penicillin every day and fed normally. The animals were sacrificed at 4 w after surgery, and the distal femur was taken and fixed in 4% paraformaldehyde for H&E, Masson and CD31 immunohistochemical staining, respectively.

## Results

### Degradation behavior of PA-MOC in vitro

**Fig. 1:**
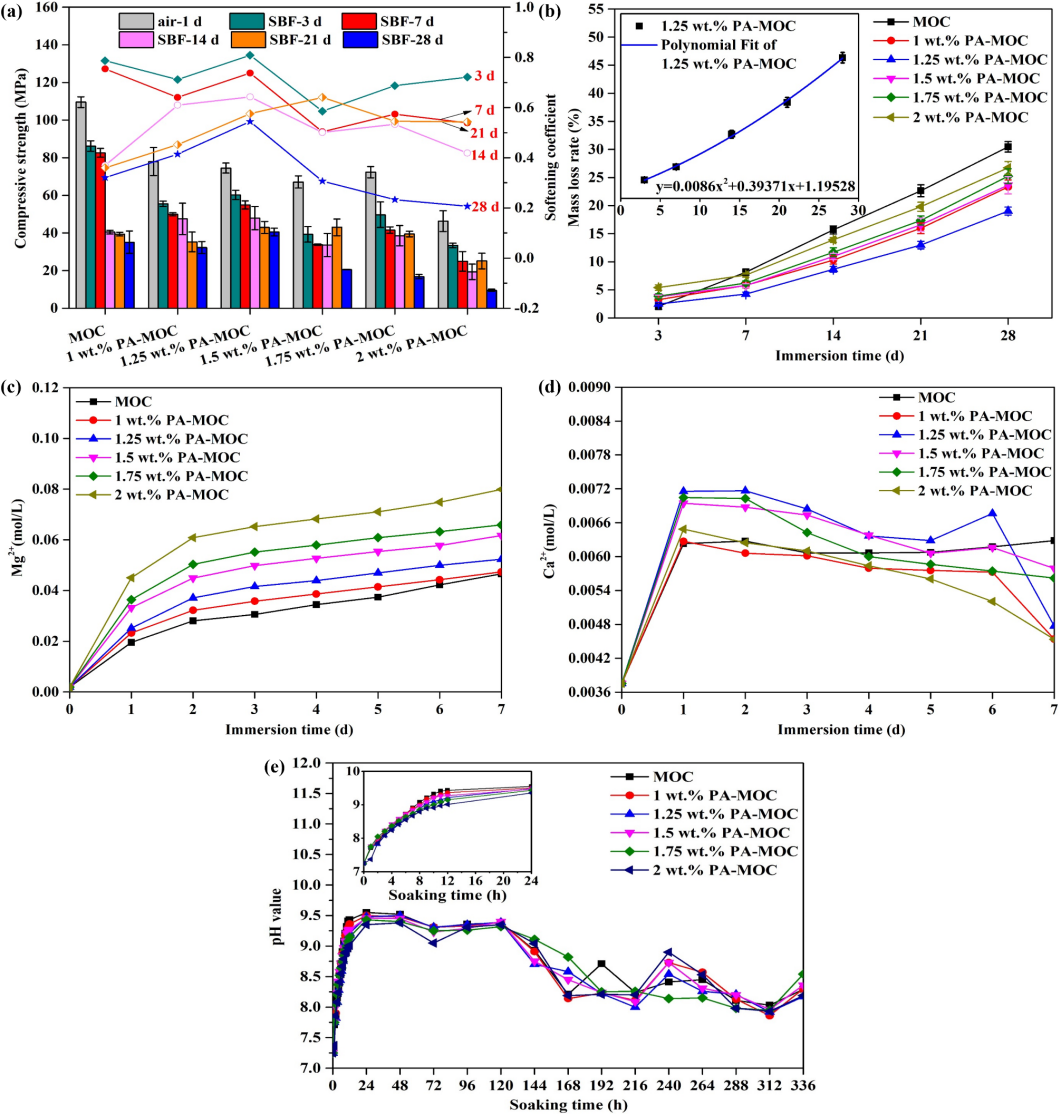
The results of in vitro degradation of PA-MOC in vitro. **a**: Compressive strength and softening coefficient of PA-MOC after curing in air for 1 d and soaking in SBF for different ages; **b**: Mass loss rate of PA-MOC after soaking in SBF for different ages; **c**: Changes of Mg^2+^ concentration (mol/L) in SBF solution; **d**: Changes of Ca^2+^concentration (mol/L) in SBF solution; **e**: pH value of PA-MOC after soaking in SBF for different ages The results of in vitro degradation of PA-MOC in vitro. **a**: Compressive strength and softening coefficient of PA-MOC after curing in air for 1 d and soaking in SBF for different ages; **b**: Mass loss rate of PA-MOC after soaking in SBF for different ages; **c**: Changes of Mg^2+^ concentration (mol/L) in SBF solution; **d**: Changes of Ca^2+^concentration (mol/L) in SBF solution; **e**: pH value of PA-MOC after soaking in SBF for different ages

Figure 1 showed the results of in vitro degradation of PA-MOC. According to the test results of compressive strength and softening coefficient of PA-MOC after curing in air for 1 d and soaking in SBF for different ages in Fig. 1(a), the initial strength of MOC decreased to varying degrees after PA was added. After soaking in SBF, the compressive strength of each group showed a decreasing trend with the extension of soaking time. The initial compressive strength of MOC was 109.5 ± 2.9 MPa, and after soaking in SBF for 28 d, the strength was 35.1 ± 5.9 MPa, and the strength loss rate was 67.9%. The initial compressive strength of MOC containing 1.25 wt% PA was 74.5 ± 2.6 MPa. After soaking for 28 d, the strength was 40.5 ± 2.0 MPa, and the strength loss rate was 45.6%.

Figure 1(b) showed the mass loss rate of PA-MOC after soaking in SBF solution for different ages. It could be seen that the mass loss rate increased with the extension of soaking time. After soaking for 28 d, the mass loss rate of MOC reached 30.5 ± 0.9%. The mass loss rate of 1.25 wt% PA in each cycle was low, and the mass loss rate of 28 d was only 19.0 ± 0.7%, indicating that 1.25 wt% was the best content of PA. According to the fitting curve, under ideal conditions, the complete degradation time of 1.25 wt% PA-MOC group was 86.7 d, which may provide good mechanical support for body tissues before the complete growth of new bone.

Figure 1(c) and (d) showed the concentrations of Mg^2+^ and Ca^2+^ after PA-MOC was soaked in SBF for different ages, the concentration of Mg^2+^ continued to increase, which was caused by the continuous release of Mg^2+^ due to the hydrolysis of the sample subjected to water erosion. The concentration of Ca^2+^ ion increased first and then decreased. The main reason was that the sample surface was relatively dry when the sample was cured and demoulded under the condition of 50% humidity. When the sample was soaked in SBF, the sample surface absorbed the water in SBF, but the deposition of Ca^2+^ had not occurred at this time. Then, with the extension of soaking time in SBF, Ca^2+^ began to deposit on the surface of the sample, so the concentration of Ca^2+^ began to decline again.

It could be seen from the change of pH value of PA-MOC soaked in SBF for 336 h in Fig. 1(e) that within 24 h of soaking, the pH value of each group increased continuously with the extension of soaking time, and gradually decreased with the increase of PA content. With the extension of soaking time in SBF, the pH values of samples in each group showed an obvious downward trend from 120 h, and maintained between 8 and 8.5 after soaking for 168 h.

### Microstructure analysis of PA-MOC

**Fig. 2:**
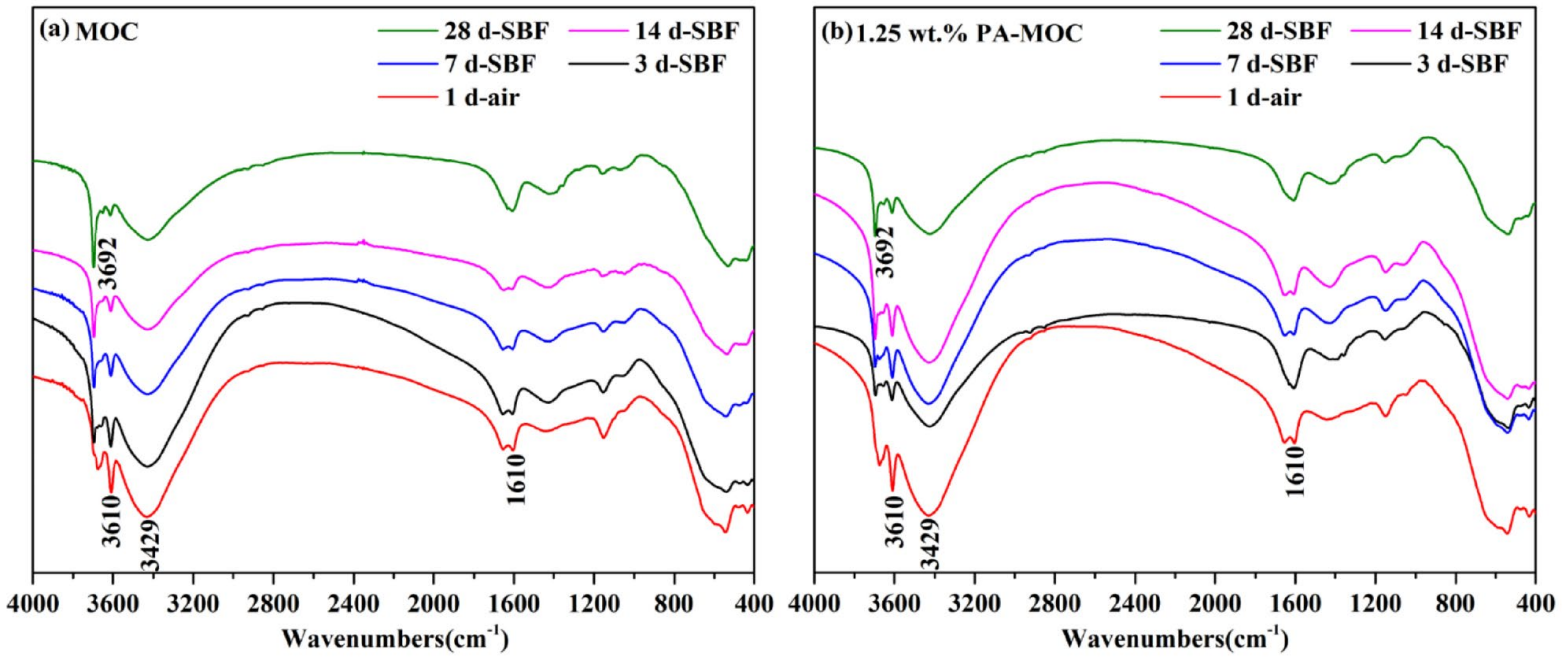
FT-IR diagram of MOC and 1.25 wt% PA-MOC after soaking in SBF for different ages

Figure 2 showed the FT-IR of MOC and 1.25 wt% PA-MOC after soaking in SBF for different ages. As can be seen from the figure, the absorption peak at 3610 cm^− 1^ was caused by the stretching vibration of non-aqueous hydroxyl group (-OH) in the phase 5 crystal and was the main characteristic peak of the phase 5 crystal in MOC [22]. The asymmetric stretching and bending vibration peaks of -OH in the phase 5 crystalline water were 3429 cm^− 1^ and 1610 cm^− 1^, respectively [22]. The absorption peak of 3692 cm^− 1^ was the stretching vibration peak of the OH in Mg(OH)_2_ [23]. With the extension of soaking time in SBF, the absorption peak strength of 3692 cm^− 1^ increased significantly, while the characteristic peak strength of phase 5 at 3610 cm^− 1^ decreased gradually, indicating that phase 5 may be hydrolyzed to Mg(OH)_2_.

**Fig. 3:**
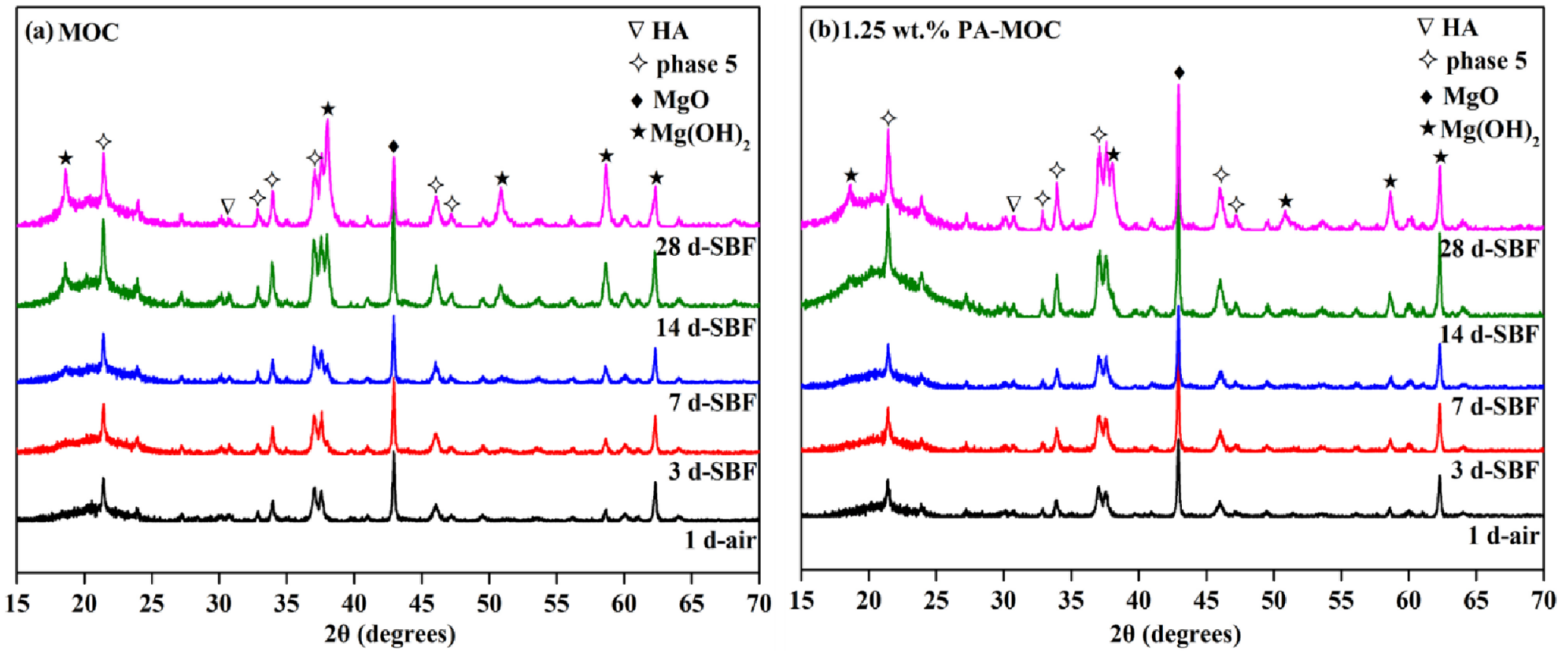
XRD patterns of MOC and 1.25 wt% PA-MOC after soaking in SBF for different ages

Figure 3 showed XRD patterns of MOC and 1.25wt. % PA-MOC after soaking in SBF for different ages. As can be seen from Fig. 3, with the extension of soaking time in SBF, the Mg(OH)_2_ peak of MOC sample gradually increased, the peak intensity of phase 5 of 1.25 wt% PA-MOC was higher, while the peak intensity of Mg(OH)_2_ was lower. The reason was that the residual MgO and phase 5 in the MOC system after soaking gradually reacted with water to form Mg(OH)_2_, and the reaction of MgO damaged the sample structure and exposed more phase 5. After the addition of PA, the hydrolysis degree of MOC was relatively low, so the intensity of the phase 5 peak in the 1.25 wt% PA-MOC structure was high. In addition, the characteristic peak of hydroxyapatite (HA) appeared in both MOC and 1.25 wt% PA-MOC samples with the extension of soaking time, and the intensity of HA peak gradually increased with the extension of soaking time, indicating that Ca^2+^ and P-containing ions in SBF had reacted on the surface of the sample to form HA crystals, among which, the HA characteristic peak of 1.25 wt% PA-MOC was relatively high, indicating that the amount of HA generated was higher.

**Fig. 4:**
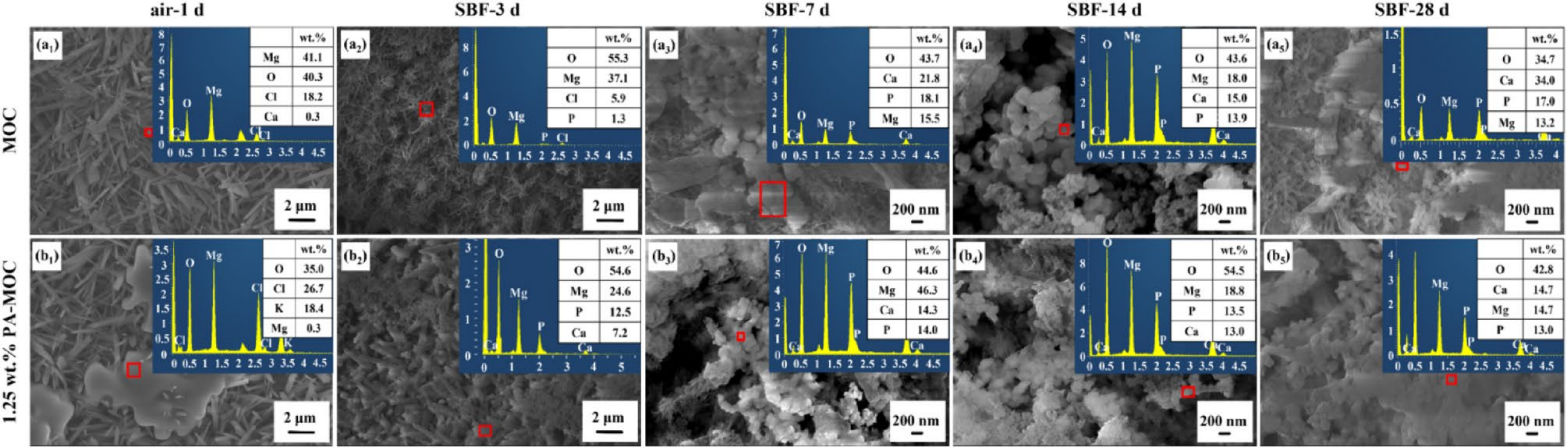
SEM images of MOC and 1.25 wt% PA-MOC after soaking in SBF for different ages

Figure 4 showed the SEM images of MOC and 1.25 wt% PA-MOC after curing in air for 1 d and soaking in SBF for different ages. Figure 4(a_1_) and (b_1_) showed the surface morphology after curing in air for 1 d. In Fig. 4(b_1_), gel-like material existed on the surface, and no spherical material deposition was found on the surface. After soaking in SBF for 3 d, the surface structure of MOC was relatively dense, so the compressive strength of MOC soaked in SBF for 3 d was higher than 1.25 wt% PA-MOC (Fig. 1(a)). After soaking in SBF for 3 d, only very small amounts of globular matter were observed (Fig. 4(a_2_)). After soaking in SBF for 7 d or more, a large number of spherical material deposits were observed on the surface (Fig. 4(a_3_) - (a_5_), (b_3_) - (b_5_)). EDS showed that the spherical material contained Ca and P elements, which suggested that the deposited spherical material was HA.

### Microscopic characterization and in vitro degradation behavior of SrR/PA-MOC

According to the above experiments, the incorporation amount of PA with better water resistance was 1.25 wt%. Therefore, the SrR was loaded on 1.25 wt% PA-MOC. The effect of SrR loading on the performance of 1.25 wt% PA-MOC samples was evaluated.

**Fig. 5:**
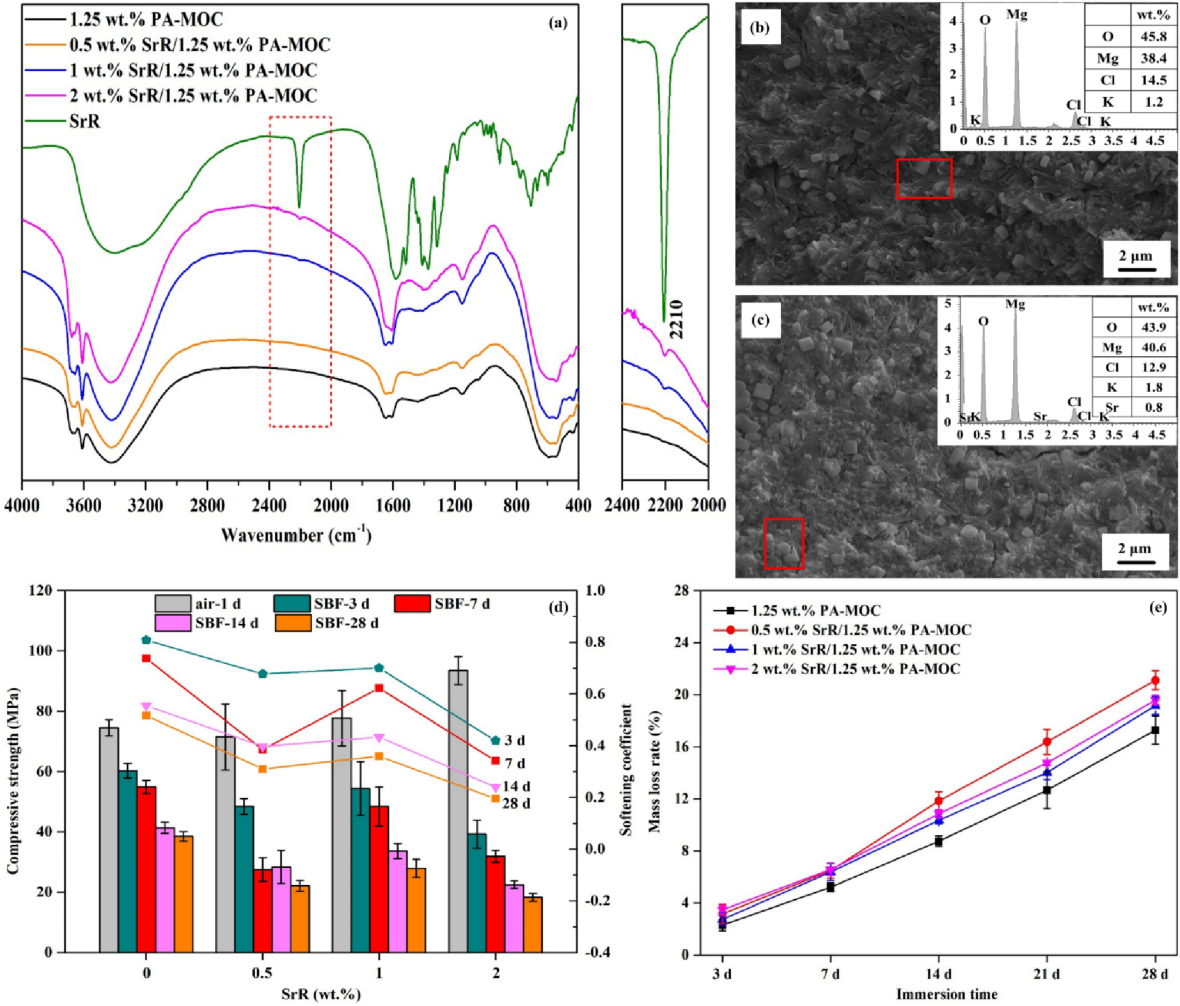
Microscopic characterization and in vitro degradation behavior of SrR/PA-MOC. **a**: FT-IR diagram of SrR/ 1.25wt. % PA-MOC after curing in air for 1 d; **b**: SEM images of 1.25 wt% PA-MOC after curing in air for 1 d; **c**: SEM images of 1 wt% SrR/ 1.25wt. % PA-MOC after curing in air for 1 d; **d**: Compressive strength and softening coefficient of SrR/1.25 wt% PA-MOC after curing in air for 1 d and soaking in SBF for different ages; **e**: Mass loss rate of SrR/1.25 wt% PA-MOC after soaking in SBF for different ages

Figure 5(a) showed the FT-IR diagram of samples loaded with SrR after curing in air for 1 d. Near 2210 cm^− 1^ was the characteristic peak of -C ≡ N contraction in SrR. The characteristic peak of 1.25 wt% PA-MOC near 2210 cm^− 1^ was consistent with that of SrR, and the peak intensity gradually increased with the increase of SrR content, indicating that SrR was successfully loaded into the sample. Figure 5(b) and (c) were the SEM images of 1.25 wt% PA-MOC and 1 wt% SrR/1.25 wt% PA-MOC after curing in air for 1 d, respectively. EDS detected Sr^2+^ in 1 wt% SrR/1.25 wt% PA-MOC sample, which proved that SrR was successfully loaded into the sample. Figure 5(d) showed the intensity variation of SrR/1.25 wt% PA-MOC. As can be seen from the results of compressive strength and softening coefficient of samples modified by SrR after soaking in SBF in Fig. 5(d), the incorporation of SrR did not significantly reduce the initial compressive strength of 1.25 wt% PA-MOC, but after soaking in SBF, with the extension of soaking time, the compressive strength of all groups decreased gradually. The compressive strength and softening coefficient of 1 wt% SrR/1.25 wt% PA-MOC were relatively high in the experimental groups modified by SrR. According to the Fig. 5(e), the mass loss rate gradually increased with the extension of soaking time. The loading of SrR all promoted the degradation of 1.25 wt% PA-MOC, and the sample group containing 1 wt% SrR had a lower mass loss rate than other mixtures, the degradation rate was slower.

### Cell experiment

**Fig. 6:**
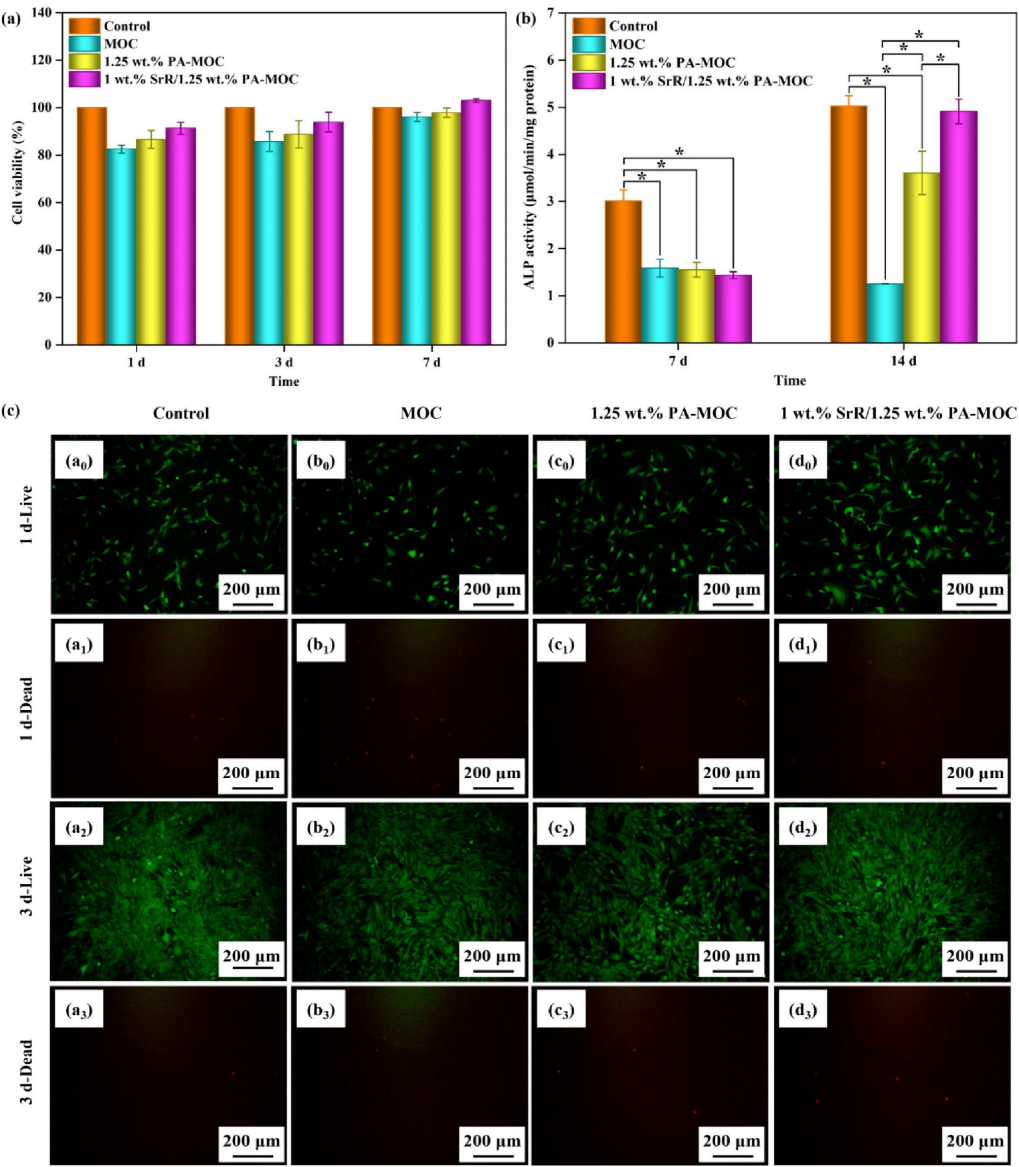
In vitro cell experiments. **a**: Cell viability of BMSCs co-cultured with extracts of MOC, 1.25 wt% PA-MOC, and 1 wt% SrR/1.25 wt% PA-MOC; **b**: ALP activity of BMSCs co-cultured with extracts of MOC, 1.25 wt% PA-MOC and 1 wt% SrR/1.25 wt% PA-MOC (There are significant differences, *p < 0.05); **c**: Live/Dead staining of BMSCs co-cultured with extracts of MOC, 1.25 wt% PA-MOC and 1 wt% SrR/1.25 wt% PA-MOC for 1 d and 3 d

Figure 6 showed the cell experiment results. As shown in Fig. 6(a), the relative cell activity of BMSCs cultured by extracts of samples tested by CCK-8 method showed that, with the extension of co-culture time with extracts of each group, the cell activity of each group showed a trend of proliferation. Compared with the blank control group, MOC, 1.25 wt% PA-MOC and 1 wt% SrR/1.25 wt% PA-MOC cells were all more than 70% active. Indicating that the extract of the sample was not cytotoxic to BMSCs (according to ISO 10993-5, cell viability results of 70% or more were considered to be non-cytotoxic [24]), the sample had good biocompatibility. Compared with MOC and 1.25 wt% PA-MOC, the cell activity value of 1 wt% SrR/1.25 wt% PA-MOC sample group was higher, indicating that the loading of SrR could promote cell proliferation, which may be related to the Sr^2+^ contained in SrR. Sr^2+^ could promote osteogenic differentiation of mesenchymal stem cells by activating Ras/MAPK signaling pathways and downstream transcription factor Runx2 [25].

ALP was a preosteogenic marker, and its activity was usually used to reflect the ability of bone cell differentiation. The influence of each group of samples on the osteogenic differentiation of BMSCs could be evaluated by ALP activity assay. As shown in Fig. 6(b), the ALP activity of BMSCs cultured with the extract of samples at 7 and 14 d showed that the ALP activity of the control group and the experimental group increased with the extension of the culture time. On the seventh day, the ALP activity of the control group was significantly higher than that of the experimental groups, and there was no significant difference among the experimental groups. On the fourteenth day, the ALP activity of 1 wt% SrR/1.25 wt% PA-MOC group was significantly higher than that of MOC and 1.25 wt% PA-MOC group, but there was no significant difference from the control group. The results showed that the addition of 1 wt% SrR could significantly improve the activity of ALP and enhance the osteogenic differentiation ability of cells, which had high application potential.

Figure 6(c) showed the staining results by Live/Dead method. Live cells were green and dead cells were red. As can be seen from the figure, the living cells showed normal spindle shape on the first day, and compared with MOC group and 1.25 wt% PA-MOC group, there were more living cells in 1 wt% SrR/1.25 wt% PA-MOC group. After culturing for 3 d, the number of living cells increased significantly. The results showed that the cell proliferation was normal and there was no obvious cytotoxicity.

### Animal experiment

#### H&E, Masson and F4/80 immunohistochemical staining of subcutaneous implanted samples

**Fig. 7:**
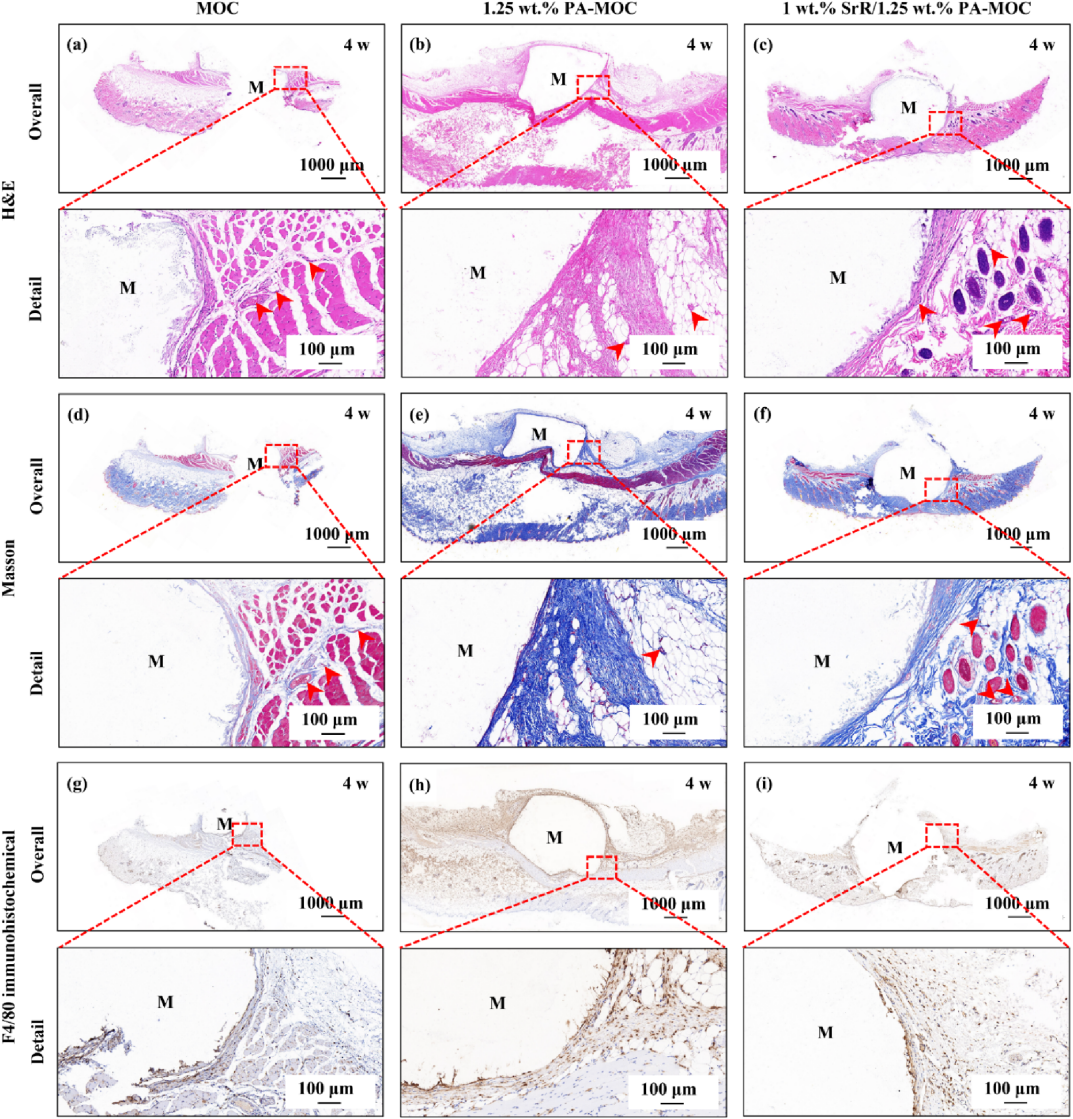
Overall and detail H&E, Masson and F4/80 immunohistochemical staining of MOC, 1.25 wt% PA-MOC and 1 wt% SrR/1.25 wt% PA-MOC after 4 w subcutaneous implantation (M: bone cement; Red arrow: blood vessel)

Figure 7 showed the H&E, Masson and F4/80 immunohistochemical staining results of MOC, 1.25 wt% PA-MOC and 1 wt% SrR/1.25 wt% PA-MOC after subcutaneous implantation for 4 w. As can be seen from the figure, the implanted material was closely combined with the surrounding tissue, and neovascularization could be observed around the material after implantation for 4 w. F4/80 was a widely used macrophage marker, and the inflammatory response of samples could be observed by labeling F4/80. As can be seen from the figure, MOC, 1.25 wt% PA-MOC and 1 wt% SrR/1.25 wt% PA-MOC samples did not cause strong immune response, and the positive expression of F4/80 cells was weak.

#### H&E, Masson and CD31 immunohistochemical staining of implanted femur samples

**Fig. 8:**
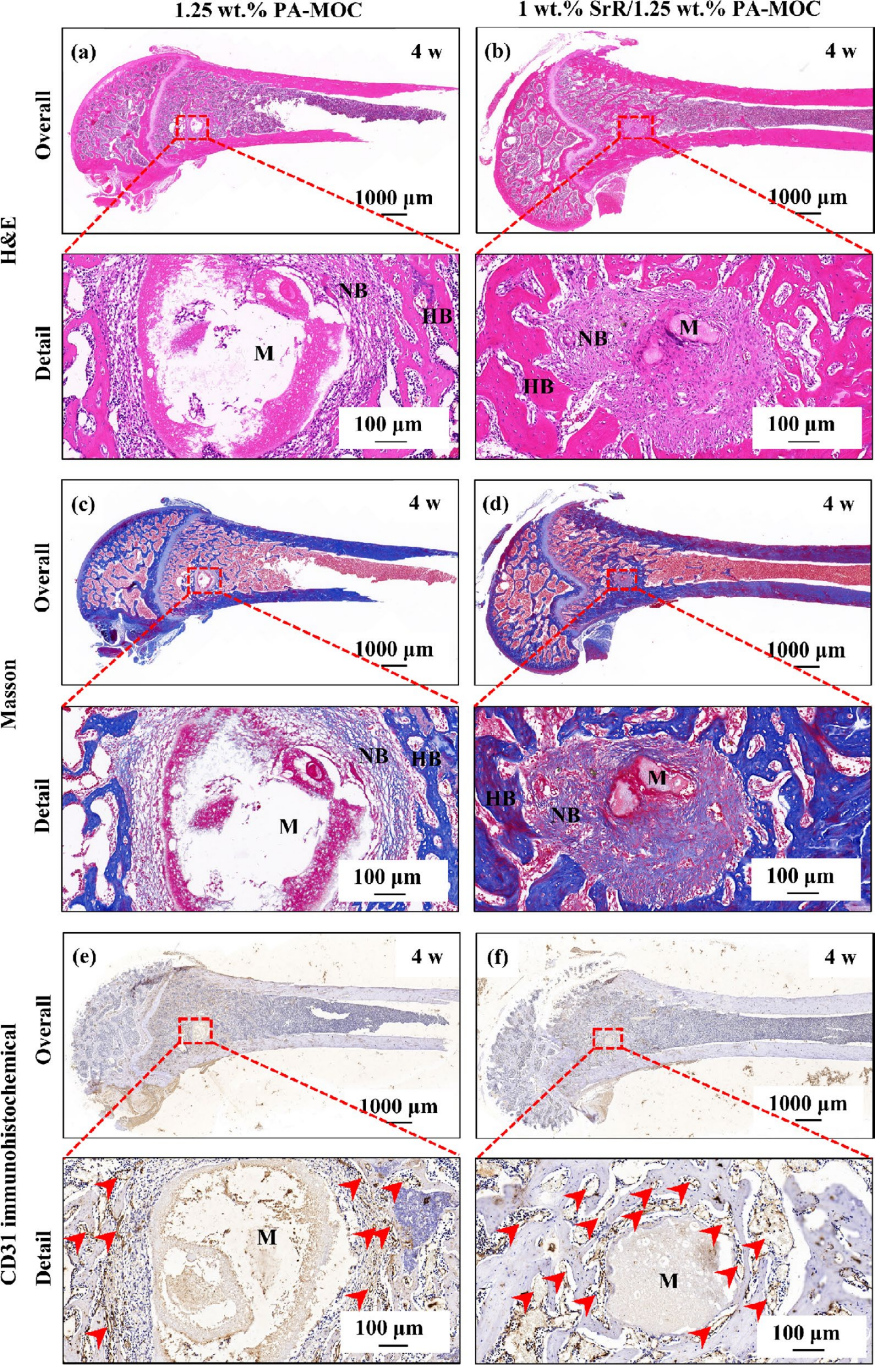
Overall and detail H&E, Masson and CD31 immunohistochemical staining at 4 w after implantation of 1.25 wt% PA-MOC and 1 wt% SrR/1.25 wt% PA-MOC in rat femur defect (M: bone cement; HB: host bone; Red hexagon area: fibrous connective tissue; Red arrow: blood vessel)

Figure 8 showed the H&E, Masson and CD31 immunohistochemical staining results of 1.25 wt% PA-MOC and 1 wt% SrR/1.25 wt% PA-MOC implanted in the femoral defect of rats for 4 w. According to H&E and Masson figures (Fig. 8(a-d)), after 4 w of implantation in rats, part of the implanted bone cement material was degraded and absorbed, and then replaced by fibrous connective tissue. Compared with the 1.25 wt% PA-MOC group, the 1 wt% SrR/1.25 wt% PA-MOC group had a higher density of fibrous connective tissue around it. CD31 was a commonly used marker to prove the existence of vascular endothelial cells. Immunohistochemical staining was used to specifically label CD31, which could be used to observe the situation of new blood vessels in tissues after implantation of different materials. As shown in Fig. 8(e-f), a large number of new blood vessels were observed after the sample was implanted into the rat femur for 4 w. This was because local tissue bleeding formed hematoma in the initial stage of bone repair. Under the action of regulatory factors in the blood clots, the fibrous connective tissue in the defect was gradually absorbed and degraded, and new bone replaced the fibrous connective tissue in the defect. This was accompanied by a large number of new angiogenesis. Compared with the 1.25 wt% PA-MOC group, the degradation of 1 wt% SrR/1.25 wt% PA-MOC in vivo was more obvious, and the number of new blood vessels around the material was significantly higher, indicating that SrR promoted angiogenesis.

## Discussion

Studies showed that the incorporation of PA could improve the water resistance of MOC, and the loading of SrR further improved the bioactivity and biocompatibility of MOC. Ye et al. [26] pointed out that gel-like phase 5 could fill pores, prevent free water from contacting with acicular phase 5, reduce the decomposition of acicular phase 5 in water, and improve the water resistance of MOC. After curing in air for 1 d, acicular phase 5 crystals were found on the surface of both MOC and 1.25 wt% PA-MOC samples. At the same time, there were a large number of gel-like phase 5 on the surface of 1.25 wt% PA-MOC sample (Fig. 4(a), (a_1_)). This was because in the hydration reaction of 1.25 wt% PA-MOC, the formation and hydrolysis of phase 5 were affected by PA. When the sample was soaked in SBF, phase 5 was hydrolyzed to Cl^−^, OH^−^ and polynuclear complex [Mg_p_(OH)_q_(H_2_O)_r_]^2p–q^, then [Mg_p_(OH)_q_(H_2_O)_r_]^2p–q^ gradually decomposed Mg^2+^. PA chelated Mg^2+^ to Mg(OH)_2_, preventing Mg^2+^ from converting to Mg(OH)_2_. It promoted the formation of PA-stable polynuclear complexes [6, 11]. Therefore, 1.25 wt% PA-MOC had higher compressive strength and softening coefficient, and lower mass loss rate (Fig. 1(a) and Fig. 1(b)). Compared with MOC, the XRD pattern (Fig. 3) showed a higher content of phase 5 in 1.25 wt% PA-MOC, while the peak intensity of Mg(OH)_2_ was lower, which was consistent with the relatively lower peak intensity of -OH in Mg(OH)_2_ at 3692 cm^− 1^ in FT-IR pattern (Fig. 2).

**Fig. 9:**
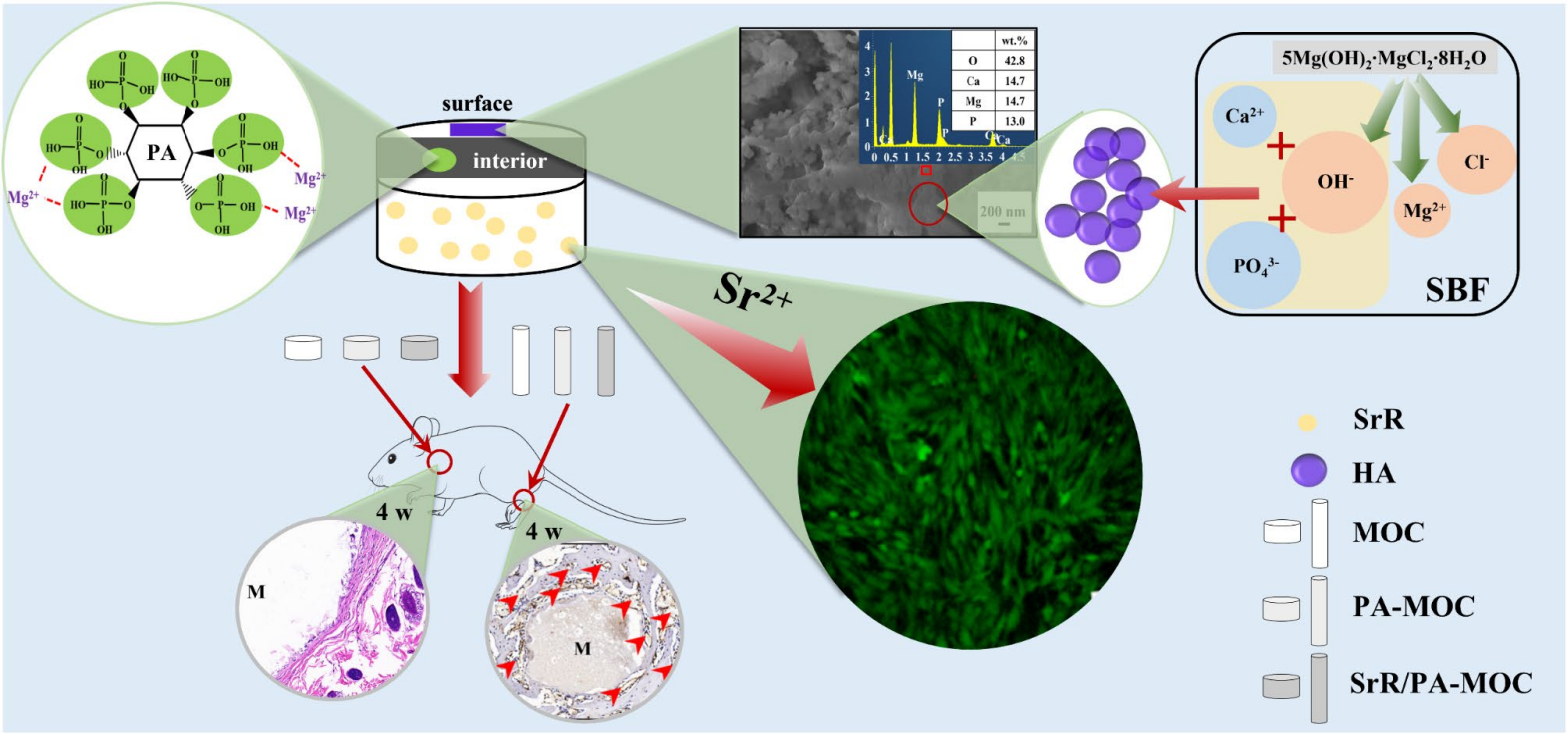
The in vitro degradation and the results of implantation in vivo

Kokubo et al. [27] proposed that biological activity of materials could be evaluated by characterizing whether HA was produced on the surface after soaking in SBF. The XRD patterns (Fig. 3) of MOC and 1.25 wt% PA-MOC soaked in SBF showed characteristic peaks of HA, and the peak intensity gradually increased with the extension of soaking time. The concentration of Ca^2+^ ions in SBF decreased gradually, and spherical substances were deposited on the surface of the sample (Fig. 1(d)), indicating that MOC could be deposited into a new phase HA after soaking in SBF, indicating that MOC had good biological activity in vitro. Figure 9 showed the in vitro degradation and the results of implantation in vivo. Studies showed that Sr^2+^ could promote the maturation and differentiation of osteoblasts by promoting the expression of osteoblast markers [28]. Sr^2+^ had dual effects, Sr^2+^ could not only promote the proliferation and differentiation of osteoblasts [28], but also inhibit the activity of osteoclasts [29]. At the same time, the slow degradation of MOC enabled the continuous release of SrR, which better prolonged the therapeutic effect of drugs and contributes to the repair of bone defects. In this study, the loading of SrR promoted cell proliferation (Fig. 6(a), (c)) and increased ALP expression (Fig. 6(b)). At the same time, a large number of new bones were generated after implantation in vivo (Fig. 8), indicating that 1 wt% SrR/1.25 wt% PA-MOC samples had good biocompatibility.

## Conclusion

In this paper, the modification of MOC was studied by PA. On this basis, SrR was loaded, and the following conclusions were obtained:

1. PA could enhance the water resistance of MOC samples in liquid environment, and the change of strength in degradation process could be regulated by regulating the content of PA. Among them, 1.25 wt% PA-MOC had a denser structure after soaking and had the best water resistance. HA was generated on the surface of MOC and 1.25 wt% PA-MOC samples, indicating it had good biological activity.
2. The extracts of MOC, PA-MOC and SrR/PA-MOC could promote cell proliferation, among which 1 wt% SrR/1.25 wt% PA-MOC had a better effect on cell proliferation, and the incorporation of SrR could improve the expression level of ALP, which was helpful to promote osteoblast osteogenesis. New bone was formed after implantation of 1.25 wt% PA-MOC into bone defect model for 4 w, indicating that 1.25 wt% PA-MOC had good bioactivity, biocompatibility and biodegradability.

The experimental results showed that PA-MOC loaded with SrR could be used as a biodegradable bone repair material and had a good application prospect in the field of biomedical materials.

## Data Availability

The data that support the findings of this study are available from the corresponding authors upon reasonable request.
